# Norovirus Cultured for the First Time

**DOI:** 10.1371/journal.pbio.0020445

**Published:** 2004-11-30

Norwalk virus and related noroviruses cause a short but unpleasant illness known variously as stomach flu, viral gastroenteritis, and winter-vomiting disease. They are the major causative agent of epidemic gastroenteritis worldwide. Norwalk virus was first described in 1968 when teachers and pupils at a school in Norwalk, Ohio, succumbed to acute gastroenteritis. The causative agent was identified in 1972, and since then, many other Norwalk-like viruses have been recognized. Noroviruses are usually picked up from contaminated food or water but can also be spread by person-to-person contact. There are no cures for norovirus infections and no vaccines. Worse still, noroviruses survive freezing, heating to 60 °C, and the amounts of chlorine added to public water supplies. Little surprise, then, that around 23 million Americans get a norovirus infection annually.

To reduce this disease burden, better prevention and control strategies for noroviruses are urgently needed. However, the development of such strategies has been hampered by the inability of scientists to find a way to grow noroviruses in cultured cells. Like all viruses, noroviruses replicate inside host cells, and they are choosy about which cells they will grow in. The discovery by Herbert Virgin and colleagues that a mouse norovirus can be grown inside dendritic cells and macrophages, two types of immune system cells, opens the door to a better understanding of norovirus biology and better disease control strategies.[Fig pbio-0020445-g001]

**Figure pbio-0020445-g001:**
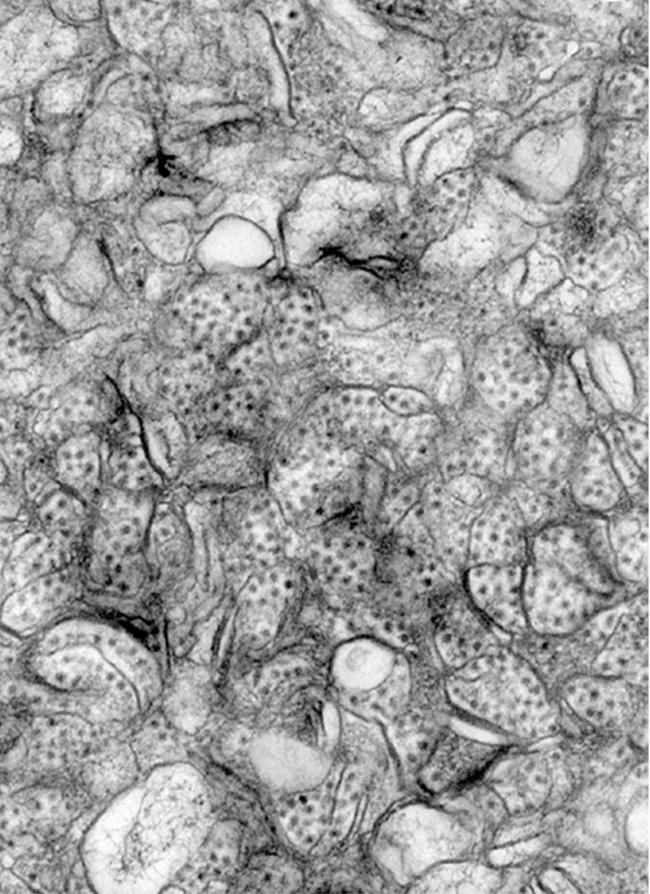
The first norovirus cell culture system

In 2003, Virgin's team discovered MNV-1, a mouse norovirus, and reported that the innate arm of the immune system (as opposed to the adaptive arm) was important in combating MNV-1 infection. The adaptive immune system involves cells that respond to a disease-causing bacterium or virus by making “adaptations” to their genomes that result in specific anti-pathogen responses. The innate immune system, on the other hand, contains cells that recognize general features of pathogens and rapidly attack them when they enter our bodies. It is our first line of defense against bacteria and viruses, and Virgin and coworkers found that mice with defective innate immune systems were particularly sensitive to MNV-1.

Now, by examining tissues taken from mice infected with MNV-1 infection, the researchers show that in live animals the virus infects macrophages (cells that engulf and kill pathogens) and dendritic cells (cells that display pathogen proteins to the adaptive immune system). This observation provided the clue needed to grow a norovirus in cultured cells for the first time: when the researchers took uninfected dendritic cells or macrophages out of animals with defective innate immune systems and grew them in the laboratory, they found that MNV-1 could replicate within these cells. The researchers then used physical and biochemical techniques to verify that what they were growing in culture was indeed MNV-1 and also determined the cellular factors that control MNV-1 growth in culture, thereby confirming that the innate immune system is important for combating norovirus infection. Analysis of the sequence of a virus attenuated by growth in vitro identified an important part of the viral capsid that plays a role in MNV-1 virulence, potentially opening up an avenue to vaccination with attenuated viruses.

The researchers speculate that dendritic cells in the gut may provide the route by which noroviruses gain access to cells deep within the lining of our guts and thus cause disease. The next step will be to see whether human noroviruses can also grow in macrophages and dendritic cells. If they do, researchers should at last be able to elucidate the lifecycle of human noroviruses and identify the cellular factors needed for their replication. Hopefully, this new knowledge will swiftly suggest ways to prevent and control the human diseases caused by noroviruses.

